# The componential processing of fractions in adults and children: effects of stimuli variability and contextual interference

**DOI:** 10.3389/fpsyg.2014.00981

**Published:** 2014-09-08

**Authors:** Li Zhang, Qiaochu Fang, Florence C. Gabriel, Dénes Szücs

**Affiliations:** ^1^Faculty of Psychology, Southwest UniversityChongqing, China; ^2^Department of Experimental Psychology, University of CambridgeCambridge, UK

**Keywords:** fractions, componential processing, holistic processing, the variability of stimuli, contextual interference

## Abstract

Recent studies have indicated that people have a strong tendency to compare fractions based on constituent numerators or denominators. This is called componential processing. This study explored whether componential processing was preferred in tasks involving high stimuli variability and high contextual interference, when fractions could be compared based either on the holistic values of fractions or on their denominators. Here, stimuli variability referred to the fact that fractions were not monotonous but diversiform. Contextual interference referred to the fact that the processing of fractions was interfered by other stimuli. To our ends, three tasks were used. In Task 1, participants compared a standard fraction 1/5 to unit fractions. This task was used as a low stimuli variability and low contextual interference task. In Task 2 stimuli variability was increased by mixing unit and non-unit fractions. In Task 3, high contextual interference was created by incorporating decimals into fractions. The RT results showed that the processing patterns of fractions were very similar for adults and children. In task 1 and task 3, only componential processing was utilzied. In contrast, both holistic processing and componential processing were utilized in task 2. These results suggest that, if individuals are presented with the opportunity to perform componential processing, both adults and children will tend to do so, even if they are faced with high variability of fractions or high contextual interference.

## INTRODUCTION

Fractions are real numbers representing continuous magnitudes through a division of two discrete integers (Meert et al., 2010). Recently, some studies have examined how adults process fraction magnitude (e.g., Bonato et al., 2007; Ischebeck et al., 2009; Kallai and Tzelgov, 2009; Meert et al., 2009; Ganor-Stern et al., 2010; Schneider and Siegler, 2010; Faulkenberry and Pierce, 2011; Sprute and Temple, 2011; Zhang et al., 2012). These studies have observed two types of processing for comparing the numerical magnitude of fractions: componential and holistic processing. When fractions are compared based on constituent numerators or denominators, it is called componential processing; when fractions are compared based on the magnitude of whole fractions, it is called holistic processing (Ischebeck et al., 2009).

A close scrutiny of previous studies suggests that the processing of fractions appears to be heavily affected by task characteristics. For fractions with different numerators and denominators, holistic processing is generally adopted (e.g., Schneider and Siegler, 2010; Faulkenberry and Pierce, 2011; Sprute and Temple, 2011). In contrast, for fractions with common components, participants primarily rely on componential processing (e.g., Bonato et al., 2007; Meert et al., 2009). Componential processing is heavily relied on and appears to take priority over holistic processing even when fractions can be correctly compared based on their holistic values or on their integer components (e.g., fractions with common numerators or denominators). The underlying reason may be that componential processing is automatic. This theory is consistent with the whole number bias (Ni and Zhou, 2005) which refers to the fact that participants use whole number knowledge to interpret fractions. Indeed, it has been shown that for unit fractions with the same numerator 1 and different denominators, the magnitude of denominators was automatically accessed both when participants were asked to process the physical size and when the numerical value was not part of the task requirements (Kallai and Tzelgov, 2009). Even for common fraction pairs without common components, the relative magnitude of denominator interfered with the processing of the whole fraction and it seems that children accessed the denominators of fractions (Meert et al., 2010).

The first objective of this study is to explore to what extent componential processing is preferred when fractions can be correctly compared based on their holistic values or on their denominators. In other words, we will explore to what extent the activation of the denominator magnitude of the fraction is automatic. Specifically, two special tasks were designed: one involving different types of fractions and the other involving high contextual interference. In both tasks, fractions can be correctly compared based on their holistic values or on their denominators. We aimed to observe whether participants prefer componential processing as a method of comparing fractions in both tasks.

The variability of fractions may be an important factor in the processing of fractions. In a study by Meert et al. (2009), Experiment 1 employed fractions with common numerators or denominators, whereas Experiment 2 also employed fractions without common components; these were used as fillers in order to increase the variability of stimuli. The results indicated that the inclusion of fillers made the task in Experiment 2 harder. Unlike that of Meert et al. (2009), this study will include unit fractions (i.e., fractions with 1 as the numerator) and non-unit fractions which are not reduced (i.e., 2/4). As such, the types of fractions are not monotonous. By including both unit fractions and non-unit fractions in the current study, we will be able to explore whether componential processing is still preferred despite the fact that componential processing can only be used after simplifying fractions.

Contextual interference may also influence the processing of fractions. In this study, when fractions alone are presented to participants, it is considered as a low contextual interference task. A high contextual interference task will be created by mixing decimals and fractions. Since the decimal system is so eminently simple, Kerslake (1991) has proposed that, once largely understood, individuals will entirely displace the clumsy system of fractions with that of decimals. Therefore, when mixing decimals and fractions, fractions might be accessed as a whole and then transformed into decimals. Furthermore, they will be interfered by decimals. In this sense, here we will consider how randomly presenting fractions and decimals (as a means of creating high contextual interference) will affect participants’ processing of fractions. Specifically, we will examine whether componential processing is preferred under the conditions of high contextual interference.

The second objective of this study is to compare the processing of fractions in children and adults. So far, there are limited studies comparing young adults and children using the same task. Gabriel et al. (2013) investigated the development of the mental representation of the magnitude of fractions; results suggested that the holistic magnitude representation of fractions is not automatically activated. Meert et al. (2010) has tested whether 10- and 12-year-olds access the magnitudes of whole fractions rather than comparing the magnitudes of their components. Results showed that children processed both types of fractions with common components holistically. Meert et al. (2010) have suggested that children are less flexible in their processing than adults. This proposition is based on the finding that adults compare numerators for fractions with common denominators and the holistic values for fractions with common numerators (Meert et al., 2009). Unlike the study by Meert et al. (2010), this study will compare both children’s and adults’ processing of fractions in the same three tasks.

Similar to previous studies (Bonato et al., 2007; Kallai and Tzelgov, 2009; Meert et al., 2009; Schneider and Siegler, 2010), in order to detect the processing of fractions, the present study is based around a classical effect in number cognition: the distance effect. The distance effect (Moyer and Landauer, 1967) refers to the increase in reaction times and decrease in accuracy that occurs as the distance between the target and the reference number decreases. The numerical distance effect has been widely observed in the literature on numerical cognition (e.g., Dehaene et al., 1993; Dehaene, 1996; Pinel et al., 2001). Thus, when the task requires a comparison between numbers, the distance effect indicates that magnitudes are compared (Bonato et al., 2007). If a distance effect that is separately related to denominators appears, it indicates a magnitude comparison between components of the fractions. That is, it indicates the componential processing of fractions. If the distance effect reflects the numerical distance between the corresponding holistic values of fractions, it indicates a magnitude comparison between the whole fractions. That is, it indicates the holistic processing of fractions. We hypothesized that even in the presence of different types of fractions and high contextual interference, componential processing is still preferred. Although both componential and holistic processing may be regarded as procedural strategies which require the use of deliberate and effortful processes, as opposed to less effortful and direct retrieval from memory (e.g., Siegler and Shipley, 1995; Campbell and Timm, 2000; Roussel et al., 2002; Geary et al., 2004), componential processing seems to be less complex compared to that of holistic. For fractions with common numerators, an inverse process is involved in componential processing. This is because larger denominators correspond with smaller fractions and hence judgments about the magnitude of denominators need to be reversed. Nevertheless, the comparison of fractions becomes easier by simply comparing denominators. For fractions with common denominators, componential processing is much easier because participants only need to simply compare numerators and no inverse process is involved. In contrast, to access the real value of a fraction, holistic processing usually requires a division operation, i.e., dividing the numerator by the denominator. Of the four basic arithmetical operations, dividing may be the most difficult to learn and the most complex to apply (Robinson et al., 2006). As Meert et al. (2009) have pointed out, except for some very common fractions (e.g., 1/2), direct retrieval of the mental magnitude of whole fractions from memory seems implausible because there are an infinite number of equivalent fractions for each ratio.

In summary, this study will explore the extent that componential processing is preferred when children and adults are faced with high variability of fractions or high contextual interference.

## MATERIALS AND METHODS

### PARTICIPANTS

Two groups of participants were recruited from one primary school and one college school in Chongqing, China. The child group consisted of primary school students from the fifth and sixth grade (*n* = 57, aged 10–12 years; female 29 and male 32), and the adult group consisted of college students (*n* = 74, aged 18–24 years; female 38 and male 36). Fifth and sixth graders were selected since children below the fifth grade had not yet learned how to compare and simplify fractions. All participants were right-handed, had normal or corrected-to-normal vision, and had no history of neurological or psychiatric illness. All participants were paid volunteers who consented to taking part in the study. This experiment was approved by the Administration Committee of Psychological Research in Southwest University and in compliance with the ethical guidelines of the American Psychological Association.

### TASKS AND STIMULI

There were three types of task. In task 1, subjects were asked to assess whether a target fraction was larger or smaller than the standard fraction (1/5). The numerator of the target fractions was 1 and the denominators varied from 1 to 9, excluding 5. In task 2, participants were asked to compare the target fractions 1/1, 2/4, 3/9, 4/16, 4/24, 3/21, 2/16, 1/9 with the standard fraction 1/5. With the exception of 1/1 and 1/9, these target fractions could be regarded as equivalent fractions of 1/2, 1/3, 1/4, 1/6, 1/7, 1/8. For example, 2/4 and 1/2 have the same values, but different integer constituents. Finally, task 3 was the same as task 2 except that the target fractions included eight decimals (0.00, 0.05, 0.10, 0.15, 0.25, 0.30, 0.35, and 0.40) in addition to the eight fractions (1/1, 2/4, 3/9, 4/16, 4/24, 3/21, 2/16, 1/9). The variables manipulated were stimuli variability (task 1 vs. task 2), contextual interference (task 2 vs. task 3), and group (children vs. adults). All variables were between-subject. Here, stimuli variability referred to the fact that fractions were not monotonous but diversiform. The stimuli variability was low in task 1 but high in task 2. Contextual interference referred to the fact that the processing of fractions was interfered by other stimuli. In task 2, the processing of fractions was not interfered by other stimuli, so this task was used as the low contextual interference task. In contrast, task 3 was used as the high contextual interference task since the processing of fractions was interfered by decimals. The dependent variables were participants’ response times (RTs) and the accuracy of fractions.

A total of 22 children and 25 adults took part in task 1, 17 children and 24 adults participated in task 2, and the remaining 18 children and 25 adults performed task 3. All children or adults were randomly allocated to one of the three tasks. There were no significant differences in terms of the average age of children [*F* (2,58) = 0.09, *p* > 0.05] or adults [*F* (2,71) = 0.51, *p* > 0.05] in the three conditions. Two numerical distances were computed for each target fraction and standard fraction: the componential distance between the denominators, and the holistic distance between the whole fractions. The componential distances were 4, 3, 2, 1, 1, 2, 3, and 4 for the eight fractions, and the holistic distances for these fractions were 0.80, 0.30, 0.13, 0.05, 0.03, 0.06, 0.08, and 0.09, respectively.

Fractions were presented as two vertically displaced digits separated by a horizontal line. These were displayed as white printed characters (Times New Roman font, normal style) on a black background. The dimensions were 12 mm × 27 mm (1.1 × 2.6). In task 3, the decimals were 27 mm × 12 mm (2.6 × 1.1). The viewing distance was approximately 60 cm.

### PROCEDURE

For each type of task, participants were required to judge whether the fraction was smaller or larger than 1/5 by pressing one of two response keys with either their left or right index finger. For tasks 1 and 2, two blocks were presented in a random order. Each block consisted of 80 trials with 10 trials for each fraction type, resulting in 160 trials overall. The assignment of response keys was counterbalanced across subjects. In one of the blocks, participants were instructed to press the “F” key if the target fractions were larger than 1/5 and to press the “J” key if the target fractions were smaller than 1/5. In the other block the response pattern was reversed. The procedure for task 3 was the same as for tasks 1 and 2 except that there were four blocks and a total of 320 trials to accommodate the additional decimal trials. The experimental procedure was controlled by a Genuine-Intel compatible 2993 MHz PC using E-prime software, 1.1 version.

The participants were tested individually in a quiet room. Tasks 1 and 2 lasted approximately 20 min and task 3 approximately 30 min. Participants were required to respond as quickly and as accurately as possible. After being presented with the task instructions, participants performed a training block consisting of eight trials. Each trial started with a fixation cross (+), which appeared at the center of the screen for 300 ms followed by a blank screen for 300–500 ms. The target fraction was then presented for 3000 ms or until participants responded. A blank screen was then shown for 1500 ms before the start of the next trial.

## RESULTS

### STATISTICAL ANALYSES

Trials with RTs shorter than 200 ms and longer than 2500 ms were excluded. Analyses were run on the accuracy and on the medians of RTs for correct responses computed for each participant and for each fraction across all trials. First, linear regression analyses with each of the distance types as predictor were separately conducted to test the effects of the holistic distance and the componential distance on median RTs and accuracy. Then, two predictors were simultaneously entered in a regression to assess their relative contribution when they were both significant, as done in the study by Meert et al. (2009). Given the moderate intercorrelation between componential and holistic distances (*r* = 0.60), the method suggested by Tzelgov and Henik (1991) could be used to detect the redundancy effect and suppression effect. Nevertheless, unlike the study by Meert et al. (2009), when only one predictor was significant, we also conducted multiple regressions because the prediction might be primarily due to the intercorrelation between the two kinds of distance. Thus, when both distances were entered into a multiple regression, neither of them was likely to be a significant predictor. If this was the case, it would suggest that both predictors accounted for the same variance (i.e., redundancy) making it impossible to assess which distance was the important factor.

For task 1, simple regressions for children revealed that both componential and holistic distances significantly predicted RTs, as listed in **Table 1**. Further multiple regression indicated that only the componential distance was a significant predictor (*R^2^* = 0.06), *B* = -29.74, SE = 13.37, *t* = -2.23, *p* < 0.05, while the effect of the holistic distance was no longer significant,*B* = -45.13, SE = 61.53, *t* = -0.73, *p* > 0.05. These results suggest that the effect of the holistic distance on RTs was suppressed and that the componential distance played a more important role. As listed in **Table 1**, however, neither the two predictors could significantly predict the accuracy rates for children.

**Table 1:** Prediction of two types of distance in Task 1.

		*R^2^*	*B*	SE	*t*	*p*
**Prediction of two types of distance on RTs**			
Children	The componential distance	0.06	-35.66	10.65	-3.35	0.00
	The holistic distance	0.04	-127.69	49.64	-2.57	0.01
Adults	The componential distance	0.06	-21.47	6.19	-3.47	0.00
	The holistic distance	0.01	-43.75	29.16	-1.50	0.14
**Prediction of two types of distance on accuracy rates**			
Children	The componential distance	0.00	0.01	0.01	0.78	0.43
	The holistic distance	0.00	0.02	0.06	0.34	0.74
Adults	The componential distance	0.07	0.01	0.00	3.76	0.00
	The holistic distance	0.02	0.02	0.01	1.85	0.07

For adults, simple regressions on both RTs and accuracy rates showed that the componential distance was the only significant predictor. Consistent with simple regressions, further multiple regression on RTs confirmed this finding (*R^2^* = 0.06), *B* = -24.73, SE = 7.76, *t* = -3.18, *p* < 0.01. In contrast, the holistic distance did not significantly predict RTs, *B* = 24.88, SE = 35.74, *t* = 0.70, *p* = 0.49. Similarly, a multiple regression on accuracy rates indicated that the effect of the componential distance remained significant (*R^2^* = 0.07), *B* = 0.01, SE = 0.00, *t* = 3.27, *p* < 0.01, while the effect of the holistic distance was not significant,*B*= -0.01, SE = 0.01, *t* = -0.46, *p* > 0.05. Together, both RTs and accuracy rates revealed that adults performed componential processing.

For task 2, simple regressions for children revealed that both componential and holistic distances could significantly predict RTs, as listed in **Table 2**. When both distances were simultaneously entered in a regression (*R*^2^ = 0.20), the effect of the holistic distance remained significant, *B* = -395.21, SE = 136.93, *t* = -2.89, *p*< 0.01, as well as the componential distance, *B* = -68.26, SE = 29.90, *t* = -2.28, *p*< 0.05. As listed in **Table 2**, the componential distance could significantly predict accuracy rates for children. However, when both distances were simultaneously entered in a regression, neither the componential distance nor the holistic distance was a significant predictor, respectively, *B* = 0.03, SE = 0.02, *t*= 1.66, *p* > 0.05, and *B*= -0.00, SE = 0.10, *t* = -0.03, *p* > 0.05, making it impossible to assess which distance was the important factor in terms of accuracy. In short, children relied on holistic processing as well as componential processing to compare fractions, as suggested by the results of RTs.

**Table 2:** Prediction of two types of distance in Task 2.

		*R^2^*	*B*	SE	*t*	*p*
**Prediction of two types of distance on RTs**
Children	The componential distance	0.15	-120.15	24.54	-4.90	0.00
	The holistic distance	0.17	-583.21	111.12	-5.25	0.00
Adults	The componential distance	0.30	-77.93	8.62	-9.04	0.00
	The holistic distance	0.22	-308.19	41.84	-7.37	0.00
**Prediction of two types of distance on accuracy rates**
Children	The componential distance	0.03	0.03	0.02	2.06	0.04
	The holistic distance	0.01	0.09	0.08	1.20	0.23
Adults	The componential distance	0.05	0.02	0.01	3.19	0.00
	The holistic distance	0.02	0.06	0.03	1.84	0.07

For adults, as revealed by simple regressions (**Table 2**), both componential and holistic distances were significant predictors of RTs. When both distances were simultaneously entered in a regression, the effects of both componential and holistic distances remained significant predictors of RTs (*R^2^* = 0.33), *B* = -59.02, SE = 10.59, *t* = -5.58, *p* < 0.001, and *B* = -144.36, SE = 48.74, *t* = -2.96, *p*< 0.01, respectively. Taken together, these results suggest that adults compared fractions by using componential processing as well as holistic processing. Simple regressions also demonstrated that both componential and holistic distances were significant predictors of accuracy rates. When both distances were simultaneously entered in a regression, however, only the componential distance significantly predicted accuracy rates (*R^2^* = 0.05), *B* = 0.02, SE = 0.01, *t* = 2.57, *p* < 0.05, but the holistic distance did not, *B* = -0.00, SE = 0.04, *t* = -0.06, *p* > 0.05. This suggests that componential processing played a more important role.

For task 3, simple regressions for children revealed both componential and holistic distances could significantly predict RTs, as listed in **Table 3**. When both distances were simultaneously entered in a regression, only the effect of the componential distance remained significant (*R^2^* = 0.14), *B* = -90.06, SE = 36.18, *t* = -2.49, *p* < 0.05, whereas the effect of the holistic distance was not significant,*B* = -282.70, SE = 165.67, *t* = -1.71, *p* > 0.05. As listed in **Table 3**, the holistic distance could significantly predict accuracy rates for children. However, when both distances were simultaneously entered in a regression, neither the holistic distance nor the componential distance was a significant predictor, *B*= 0.15, SE = 0.10, *t* = 1.41, *p* > 0.05, and *B* = 0.01, SE = 0.02, *t* = 0.51, *p* > 0.05, respectively. In short, children mainly used componential processing, as revealed by the results of RTs.

**Table 3:** Prediction of two types of distance in Task 3.

		*R^2^*	*B*	SE	*t*	*p*
**Prediction of two types of distance on RTs**			
Children	The componential distance	0.12	-127.23	29.081	-4.38	0.00
	The holistic distance	0.10	-531.01	134.70	-3.94	0.00
Adults	The componential distance	0.27	-81.39	9.44	-8.62	0.00
	The holistic distance	0.13	-255.97	47.64	-5.37	0.00
**Prediction of two types of distance on accuracy rates**			
Children	The componential distance	0.02	0.03	0.02	1.72	0.09
	The holistic distance	0.03	0.18	0.08	2.21	0.03
Adults	The componential distance	0.12	0.04	0.01	5.09	0.00
	The holistic distance	0.03	0.09	0.04	2.55	0.01

For adults, as revealed by simple regressions (**Table 3**), both componential and holistic distances were significant predictors of RTs and accuracy rates. When both distances were simultaneously entered in a regression, only the componential distance significantly predicted RTs (*R^2^* = 0.28), *B*= -75.21, SE = 11.84, *t* = -6.35 *p* < 0.001, but the holistic distance did not, *B* = -47.18, SE = 54.53, *t* = -0.87, *p* > 0.05. Similarly, only the componential distance significantly predicted accuracy rates (*R^2^* = 0.12), *B* = 0.04, SE = 0.01, *t* = 4.35, *p* < 0.001. In contrast, the effect of the holistic distance on accuracy rates was no longer significant, *B* = -0.02, SE = 0.04, *t* = -0.50, *p* > 0.05. In sum, these results indicate componential processing by adults.

In addition, in order to compare the performance of children and adults in task 1 and 2, ANOVAs on RTs and accuracy rates for each fraction with groups (children and adults) and tasks (1 and 2) as between-subject variables were conducted. The RTs and accuracy of each fraction in tasks 1 and 2 are listed in **Table 4** and **Table 5**, respectively. Results on RTs and accuracy showed significant effects of groups in all fractions, *Fs* > 24.68, *ps*< 0.001, suggesting that children performed worse than adults with longer RTs and lower accuracy. Significant effects of tasks on RTs were also seen in each fraction, *Fs* > 17.49, *ps*< 0.001, indicating that subjects responded more slowly in task 2 compared to task 1. For analyses on accuracy rates, significant effects of tasks were observed in some fractions including 1/3, 1/4, 1/6, 1/7, 1/8, *Fs* > 17.49, *ps*< 0.001, but not in the fractions 1/1, 1/2, and 1/9 (*ps*> 0.16). In addition, interactions in all fractions were significant, *Fs* > 6.45, *ps*< 0.02, except for the fraction 1/4 (*p* = 0.078). The interactions were due to the fact that the differences between RTs in tasks 1 and 2 were bigger for children than for adults in all fractions except for 1/4.

**Table 4:** The average RTs and accuracy for each fraction in task 1.

		RTs(ms)		ACC	
Group	Stimuli	*M*	SE	*M*	SE
**Children**	1/1	652	19.63	0.83	0.04
	1/2	656	23.77	0.83	0.04
	1/3	717	38.29	0.83	0.04
	1/4	774	40.00	0.81	0.04
	1/6	792	43.86	0.79	0.04
	1/7	702	34.65	0.83	0.04
	1/8	699	32.75	0.84	0.04
	1/9	697	30.96	0.83	0.04
**Adults**	1/1	539	15.39	0.99	0.00
	1/2	537	17.47	1.00	0.00
	1/3	566	22.54	0.99	0.01
	1/4	591	20.33	0.97	0.01
	1/6	607	26.83	0.95	0.01
	1/7	550	16.60	0.98	0.01
	1/8	542	20.29	0.99	0.01
	1/9	527	15.74	0.98	0.01

**Table 5:** The average RTs and accuracy for each fraction in tasks 2.

		RTs(ms)		ACC	
Group	Stimuli	*M*	SE	*M*	SE
**Children**	1/1	795	35.85	0.75	0.05
	2/4	1028	68.93	0.73	0.05
	3/9	1203	70.48	0.69	0.05
	4/16	1200	76.82	0.62	0.06
	4/24	1365	90.53	0.65	0.05
	3/21	1230	93.05	0.69	0.04
	2/16	1173	73.68	0.72	0.06
	1/9	1043	85.31	0.71	0.05
**Adults**	1/1	569	13.17	0.96	0.02
	2/4	648	23.44	0.96	0.02
	3/9	742	28.74	0.93	0.03
	4/16	871	26.19	0.87	0.03
	4/24	868	41.80	0.90	0.02
	3/21	722	19.78	0.98	0.02
	2/16	751	25.71	0.93	0.02
	1/9	672	21.26	0.96	0.01

Likewise, in order to compare the performance of children and adults in task 2 and 3, ANOVAs on RTs and accuracy rates for each fraction with groups (children and adults) and tasks (2 and 3) as between-subject variables were conducted. The RTs and accuracy of each fraction in task 3 can be seen in **Table 6**. Results on RTs and accuracy showed significant effects of groups in all fractions, *Fs* > 16.28, *ps*< 0.001, suggesting that children performed worse than adults with longer RTs and lower accuracy. Significant effects of tasks on RTs were found in three fractions including 1/1, 2/4, and 4/16, *Fs* > 4.92, *ps* < 0.03, in which participants responded slowly in task 3 as compared to in task 2. No interactions were significant in RT and accuracy analyses (*ps* > 0.09).

**Table 6:** The average RTs and accuracy for each fraction in task 3.

		RTs(ms)		ACC	
Group	Stimuli	*M*	SE	*M*	SE
**Children**	1/1	961	45.46	0.79	0.05
	2/4	1168	77.06	0.75	0.05
	3/9	1288	119.33	0.71	0.05
	4/16	1440	123.45	0.68	0.05
	4/24	1427	80.94	0.69	0.05
	3/21	1306	79.46	0.71	0.06
	2/16	1224	105.71	0.73	0.06
	1/9	1195	105.47	0.70	0.07
**Adults**	1/1	643	14.37	0.95	0.01
	2/4	726	29.51	0.95	0.02
	3/9	819	34.42	0.88	0.03
	4/16	957	36.60	0.77	0.04
	4/24	872	35.70	0.90	0.02
	3/21	774	25.60	0.93	0.02
	2/16	786	34.75	0.94	0.02
	1/9	671	15.62	0.94	0.02

## DISCUSSION

This study showed that stimuli variability and contextual interference significantly influenced the processing of fractions in both adults and children. The RT results suggest that adults and children process fractions in a very similar way. Only componential processing was used in task 1 and task 3. In contrast, holistic processing as well as componential processing were adopted in task 2. It seems that the variability of fractions influenced the processing of fractions, leading both adults and children to resort to holistic processing. Interestingly, however, despite the variability of fractions, when fractions were interfered with decimals, both children and adults preferred componential processing.

In task 1, which just involved unit fractions, both adults and children only used componential processing. However, as revealed by the RT results, both componential and holistic processing were adopted by children and adults in task 2 which involved different types of fractions. This finding indicates that both the magnitude of the whole fraction and the denominators of fractions can be accessed. Compared to task 1, task 2 used irregular non-unit fractions to increase the variability of fraction stimuli. The irregular non-unit fractions in task 2 were less familiar than those in task 1 (Ganor-Stern, 2012), although their holistic values were equal. Previous studies have indicated that the stimuli format can affect numerical processing (e.g., Campbell et al., 2004; Campbell and Penner-Wilger, 2006). For example, Campbell et al. (2004) showed that an unfamiliar stimuli format disrupted number-fact retrieval and promoted the use of more effortful procedural strategies. It is therefore understandable that holistic processing was adopted when fractions were relatively irregular and unfamiliar since this processing strategy is both more stable and more likely to produce a correct answer in spite of complexity.

It is very interesting that componential processing was not abandoned in task 2 despite the fact that holistic processing would have ensured correctness. The use of componential processing in task 1 is easily understood since the denominator of the target fraction could be directly compared with that of the standard fraction. However, the same strategy could not be adopted in task 2, wherein subjects had to simplify most of the target fractions and keep these simplified fractions in mind (particularly their denominators) in order to arrive at an answer for fraction comparison. It may reflect the fact that componential processing is very automatic for both adults and children.

It seems that the preference for componential processing was not affected by contextual interference. For both adults and children, the holistic distance effect was significant with low contextual interference in task 2 but not with high contextual interference in task 3. This finding indicated that holistic processing was abandoned under high contextual interference and participants mainly depended on componential processing in task 3. It seems that, when faced with the option of processing stimuli componentially, adults and children always prefer to process fractions in this way. As stated before, the possible reason for this is that the access to numerical magnitudes of denominators for fractions with common numerators is direct and automatic.

As revealed by Kallai and Tzelgov’s (2009) study, when numerical processing was not necessary for the task, denominators of unit fractions with the same numerators were processed automatically. In contrast, slower and more complex holistic processing is likely to require more working memory resources. However, the high contextual interference task also requires some working memory resources in order to suppress the interference from decimals; this might leave insufficient resources for holistic processing. It is perhaps for this reason that subjects mainly depended on componential processing which is relatively fast and less effortful. It should be noted that participants responded slowly to1/1, 2/4, and 4/16 in task 3 compared to those in task 2. These fractions are very familiar and are easily written as finite decimals, so it is possible that their real values wereactivated at the same time as their components were being automatically accessed. Consequently, since their real values needed to be inhibited, more working memory resources are likely to have been activated for the processing of these three fractions. Accordingly, this may account for the longer RTs in task 3 relative to task 2.

Taken together, adults and children show a similar preference for the componential processing of fractions based on the RT results. However, there are also some differences between adults and children in terms of their processing of fractions. Based on the results of accuracy rates, adults relied on componential processing in all the three tasks. However, children did not show any evidence for componential processing. This finding may reflect the fact that adults are better equipped to adopt componential processing than children. Indeed, children performed worse than adults with longer RTs and lower accuracy in all the three tasks.

In conclusion, if faced with the opportunity to process fractions componentially both children and adults tend to show a preference for componential processing even when there is a variability of fractions and high contextual interference. However, when there was a variability of fractions, both adults and children resorted to holistic processing in addition to componential processing.

## Conflict of Interest Statement

The authors declare that the research was conducted in the absence of any commercial or financial relationships that could be construed as a potential conflict of interest.
